# Can models of percutaneous absorption based on in vitro data in frogs predict in vivo absorption?

**DOI:** 10.1371/journal.pone.0235737

**Published:** 2020-07-29

**Authors:** Victoria K. Llewelyn, Lee Berger, Beverley D. Glass

**Affiliations:** 1 Pharmacy, College of Medicine and Dentistry, James Cook University, Townsville, Queensland, Australia; 2 One Health Research Group, Melbourne Veterinary School, University of Melbourne, Werribee, Victoria, Australia; Auburn University College of Veterinary Medicine, UNITED STATES

## Abstract

The primary aim of in vitro testing of chemicals delivered via the percutaneous route is to predict the absorption that would ensue if exposure occurred in live animals. While there is mounting evidence that in vitro diffusion studies in mammalian skin can provide valid information regarding likely in vivo absorption, little is known whether such a correlation exists between in vitro diffusion testing and in vivo blood levels in amphibians. The current study used previously-reported in vitro absorption data for caffeine, benzoic acid, and ibuprofen across isolated skin from the cane toad (*Rhinella marina*) to produce a series of linear mixed-effect models of the absorption parameters flux and permeability coefficient (K_p_). Models investigated the relative impacts of animal weight, physicochemical characteristics of the applied chemical (logP or molecular weight), and site of application. The top models were then used to predict the flux, K_p_ and serum concentrations of the same three model chemicals. Finally, the absorption of these chemicals was determined in live cane toads, and results compared to the model predictions. LogP and site of application were included in all top models. In vivo absorption rates were lower than predicted for all chemicals, however, the models provided reasonable predictions of serum concentration, with factors of difference (FOD) ranging from 2.5–10.5. Ibuprofen, the chemical with the highest relative lipophilicity, had the poorest predictive performance, consistently having the highest FOD for all predictions. This report presents the first models of percutaneous absorption in an amphibian. These models provide a basic method to establish the approximate in vivo absorption of hydrophilic and moderately-lipophilic chemicals through frog skin, and could therefore be used to predict absorption when formulating such chemicals for treatment of disease in frogs, or for risk-assessments regarding chemical pollutants in frog habitats.

## Introduction

In vitro methods are commonly used in place of in vivo experimentation to predict the percutaneous absorption of chemicals for both drug development and risk assessment. The diffusion cell, an apparatus that holds either excised skin or a synthetic membrane between two glass chambers, is the most commonly-used and recommended in vitro technique to measure drug penetration for both development and equivalence testing of therapeutic formulations, and dermal exposure risk assessments [1, 2]. However, in vitro methods need to be validated by comparison to the in vivo situation, to assess their relevance and application to practice.

Percutaneous absorption studies in human and other mammalian species have found moderate correlation between in vitro methods and results in vivo, provided that study methods are sufficiently harmonized with regard to donor composition (vehicle and dose), skin source, occlusion of the skin, exposure time, receptor fluid composition, and diffusion area [3–6]. However, until recently no established guidelines have specifically addressed in vitro-in vivo correlation for transdermal drug products, and much discrepancy still remains with regards to harmonisation between studies. The European Medicines Agency has attempted to rectify this, recently releasing draft guidelines to advise in vitro-in vivo correlation for transdermal drug products for consultation [7].

Amphibians have highly permeable skin, providing an ideal surface for absorption of chemicals. However, relatively few studies exist that investigate the absorption kinetics of chemicals through frog skin (for review, see [8]). Recent risk assessment guidelines have highlighted the detrimental impact of this heightened permeability coupled with lack of absorption kinetics studies on frog health, and have recommended 100% absorption be assumed for all chemicals through frog skin until further research has been conducted [9]. While there are several studies that report on the absorption of chemicals in frog skin in vitro [10–17], only Riviere, Shapiro [11] and Roberts, Berger [17] have also reported on in vivo absorption for their investigated chemicals. Both studies reported lower in vivo drug levels than those found in the concurrent in vitro studies, however the magnitude of difference varied, with Riviere, Shapiro [11] reporting serum concentrations of gentamycin 9–12-fold lower than those predicted based on the in vitro studies, while Roberts, Berger [17] reported terbinafine concentrations of 0.22 μg/mm^2^ following 5 hours exposure in vitro, and 0.1 μg/mm^2^ following 4 hours exposure in vivo. Owing to the conflicting results of these two studies, and the lack of other studies reporting both in vitro and in vivo absorption in frog skin, it remains to be ascertained whether the remaining reports of in vitro absorption through frog skin [10–17] can be reliably translated to the in vivo situation.

The current study builds on previous work by the authors to determine how closely in vitro absorption studies in frogs correlate with the in vivo situation. Firstly, previously-collected in vitro percutaneous absorption data [13] were used to produce a model of percutaneous absorption in the cane toad (*Rhinella marina*). The developed model was then used to predict the in vivo absorption of a series of model chemicals, and finally the absorption of the same chemicals was measured in healthy adult cane toads, and results compared.

## Materials and methods

### Chemicals and solutions

Model chemicals were reagent grade caffeine, ACS reagent grade benzoic acid (both Sigma-Aldrich) and ≥98% ibuprofen (Sigma). Amphibian Ringer’s solution (ARS) was prepared according to [18]: 113 mM sodium chloride, 2 mM potassium chloride, 1.35 mM calcium chloride, 2.4 mM sodium bicarbonate. Model drug solutions were prepared as described previously by the authors for in vitro absorption studies in cane toads [13]: a saturated solution for each of the model chemicals was prepared in ARS, with ibuprofen solutions also containing 2.75 mg/mL 2-hydroxypropyl-beta-cyclodextrin (HPβCD; Aldrich Chemistry) to ensure adequate solubilization of ibuprofen. Ethyl 3-aminobenzoate methane sulfonate (MS-222; Aldrich Chemistry) was prepared as a 0.2% w/v bathing solution in purified water, buffered to pH 7.3 with sodium bicarbonate, or as a 12.5% w/v injection in purified water, unbuffered [19].

Serum extractions used either hydrochloric acid (analytical grade; Ajax Finechem PTY LTD) and dichloromethane (high-performance liquid chromatography (HPLC) grade; Burdick & Jackson) or ethyl acetate (reagent grade; Ajax Finechem PTY LTD). HPLC mobile phase was methanol (HPLC grade; Fisher Chemicals, Trinidad and Thermo Fisher Scientific, Australia) and ultrapure water (Milli-Q Integral, Millipore Australia), acidified with 0.2% v/v acetic acid (analytical grade; RCI LabScan, Thailand). Water used throughout was ultrapure (Milli-Q Integral, Millipore Australia). All solutions were freshly prepared on the day of use.

### Animal husbandry

Adult male cane toads (*Rh*. *marina*) were wild-caught in the Townsville region (Australia) and transported to the laboratory, where they were housed in a dedicated room maintained at 21±2°C. Ninety-two animals were used for the study, weighing 71.61 g–138.16 g (mean 98.48 g). Animals were housed in plastic tubs (60 x 36 x 40 cm); each tub had a lid with holes to permit airflow, and had its interior base lined with absorbent paper. Three or four animals were housed in each tub, which also provided two retreat sites and a water dish. Water was provided ad libitum, and toads were fed crickets dusted with calcium and multivitamin powder (Vetafarm Herpevet Multical Dust) every 2–3 days, with all toads fed for the last time two days before the study commenced. Animals were housed for at least five days prior to testing, to allow for acclimation to their surrounds. All animal handling, husbandry, and experimental methodology was approved by the James Cook University Animal Ethics Committee (A2551).

### In vivo study

Prior to the study commencing, each animal was rinsed with ARS to ensure the skin was clean, and animal weight was recorded. Animals were then transferred to individual plastic containers for commencement of the trial.

For each individual chemical trial, 25 mL of saturated chemical solution was transferred to a plastic zip-lock bag. Each animal was transferred to an individual bag and each bag placed in an individual plastic container that restricted movement, ensuring relatively consistent exposure of the ventral pelvic skin region to the chemical solution. In contrast to previous in vitro studies by the authors [13] where chemical application was measured specifically in skin samples from dorsal, ventral thoracic and ventral pelvic, isolating aqueous drug solution application to only the dorsal or ventral thoracic skin surfaces is impossible in live frogs. As frogs naturally sit with their pelvic ventrum in small bodies of water (e.g., puddles), application to this region is relatively easy, and mimics real-world conditions.

Animals were randomly allocated to a chemical treatment and sampling time using random number generator software. Samples were obtained at t = 0, 30, 60, 90, 120, 150, 180, 240, 300, 360 minutes for benzoic acid, t = 0, 30, 60, 90, 120, 180, 240, 300, 360 minutes for ibuprofen, and t = 5, 10, 15, 20, 25, 30 minutes for caffeine. Exposure and sampling times for benzoic acid and ibuprofen were selected to match those used in previous in vitro studies. Sampling times for caffeine were revised following a preliminary trial which suggested restlessness in animals exposed to caffeine for more than 30 minutes. For each chemical trial, four animals were sacrificed at each sampling time, in accordance with dermal absorption guidelines [20].

At each sampling time, animals were removed from their bathing solution and rinsed in fresh purified water. Each toad was then anaesthetised by intracoelomic injection of 400 mg/kg MS-222 [19], and once a deep plane of anaesthesia was achieved, the thoracic cavity was opened and 1 mL of cardiac blood was removed via a heparinised capillary tube into an Eppendorf tube. A deep plane of anaesthesia was achieved within 2 minutes of administration of MS-222 for all animals. Following sample collection, each animal was euthanized by placing in a bath of 0.2% w/v MS-222, buffered with sodium bicarbonate. Blood samples were allowed to clot, then centrifuged at 12,000 RCF for 10 minutes to separate serum. 0.5 mL aliquots of serum were then transferred to Eppendorf tubes, and were stored at –80°C until analysis.

### Sample extraction

Extraction of ibuprofen and benzoic acid was based on the method described by Kearns and Wilson [21]. Briefly, 0.2 mL of 5M hydrochloric acid was added to 0.5 mL thawed serum, the mixture was vortexed for 60 seconds, and 3 mL dichloromethane added. The resultant mixture was vortexed for a further 2 minutes, and then centrifuged at 3,200 RCF for 5 minutes at 4°C. The organic layer was then transferred to a clean glass tube and gently dried under nitrogen gas at 35°C. Evaporated samples were reconstituted with 1 mL of mobile phase (60% methanol in water with 0.2% acetic acid), vortexed and then centrifuged a final time for 5 minutes. The clear supernatant was transferred to a sample vial and transported to the analytical laboratory for analysis.

For extraction of caffeine, 7 mL of ethyl acetate was added to each thawed 0.5 mL aliquot of serum. The sample was then vortexed for 60 seconds and centrifuged at 3,200 RCF for 5 minutes at 22°C. Supernatant was then transferred to a clean glass tube and dried overnight at 37°C in a rotary dryer. Evaporated samples were reconstituted with 1 mL of mobile phase, vortexed and then centrifuged a final time at 3,200 RCF for 5 minutes. The clear supernatant was transferred to a sample vial and samples transferred to the analytical laboratory for analysis. Preliminary studies indicated that 98% of caffeine was present following a single extraction, and so further extractions were not performed.

### Ultra-high-performance liquid chromatography (UHPLC)

Analysis of serum samples was performed on a Shimadzu UHPLC Nexera X2, with an SPD-M30A Diode Array Detector. Post-run analysis was performed using Labsolutions 5.89 (Shimadzu). Separation was achieved on an Applied Biosystems SPHERI-5 5 Micron ODS column (250 x 4.6 mm) at 38°C. The mobile phase was a gradient with a total run time of 15 minutes, consisting of 0.2% v/v acetic acid in water and 0.2% v/v acetic acid in methanol, as follows: 0–12 minutes increasing from 50 to 85% methanol, remaining at 85% methanol for the final 3 minutes. Flow rate was 1 mL/min. Injection volumes were 50 μL for ibuprofen and benzoic acid, and 10 μL for caffeine. Quantification was at 242 nm for all chemicals. All samples were analysed in duplicate.

Calibration curves were prepared by spiking working standards of each chemical in mobile phase, as preliminary studies in pooled blank toad serum showed no matrix effects. Final concentration ranges for each chemical were as follows: benzoic acid: 0.25–62.4 μg/mL, ibuprofen: 0.3–6 μg/mL and caffeine: 0.195–200 μg/mL. All curves were linear with r^2^>0.999 for all runs. Limit of quantification for each chemical was the lowest standard. The precision of the method was assessed by injecting ten individual samples of a mid-range standard solution for each chemical. Relative standard deviation of these injections was <1% for each chemical.

### Data analysis and statistics

#### Developing a model of in vitro absorption for cane toads

Percutaneous absorption data for the models were collected from a previous paper by the authors [13]. This data included flux of three model chemicals (caffeine, benzoic acid, and ibuprofen) across isolated cane toad skin as tested using Franz diffusion cells. All data exploration and analyses were performed in R [22]. Mean permeability coefficient (K_p_) was calculated by dividing flux by the concentration of chemical in the donor solution (K_p_ = J_ss_/C_v_). Concentration of drug in the donor solution (C_v_) was the saturation solubility of each chemical in ARS, as reported in Llewelyn, Berger [13]. Separate linear mixed-effects models with different combinations of predictor variables and interactions between predictor variables were created to determine the relationship between absorption (flux or K_p_) and skin region of application, animal weight, and physicochemical properties of the applied chemical [logP or molecular weight (MW)]. As data included three chemicals, MW and logP could not both be included as covariates in the same model, and so their influence was investigated individually. Skin region was categorical and included three factor levels: dorsal, ventral thoracic and ventral pelvic. Models using both standard and logarithm-transformed values of K_p_ and flux were developed and compared. Linear mixed-effects models were fitted by maximum likelihood using the lme function in the nlme package [23]. All models included the individual animal as a random effect, and models that allowed and did not allow for heteroskedasticity in data were examined. Models were compared using ANOVA, Akaike’s Information Criterion, and examination of residual plots to determine best fit. The top models, defined as those with the largest statistical significance from the null model while being not significantly different from each other (p>0.05), were then examined for trends in independent variables. The best model, determined through a combination goodness-of-fit analyses and parsimony, was then re-fitted using restricted maximum likelihood to obtain the coefficients used to predict chemical flux for each model chemical and skin region for comparison to in vivo values determined in this study.

#### Analysis of in vivo data

In order to allow direct comparison of predictions from in vitro data and in vivo results, the average absorption per cm^2^ of skin surface area (SA) exposed to the bathing solution was calculated. As the nominal amount of bathing solution used in the study was chosen to provide drug exposure to the ventral pelvis only, skin SA for each individual was estimated using the equation of Tracy [24], which states that the SA of a frog’s ventral pelvis in contact with substrate when in a sitting position can be calculated from total body mass as:

$${SA}_{pelvic\;ventrum}=1.15{*[body\;mass(g)]}^{0.559}$$

Mean cumulative absorption versus time plots were produced for each chemical. Mean flux (μg/cm^2^/h) was determined for each chemical from the steady-state slope of the cumulative absorption versus time plot, and K_p_ was calculated as outlined above. Resultant in vivo flux and K_p_ were compared to those predicted from the top in vitro flux models for cane toads. Serum concentrations were compared to those predicted from the top in vitro models. Factor-of-difference (FOD) was calculated as the ratio between the in vitro-based model predictions and the observed in vivo values.

## Results

### Models for in vitro absorption in cane toads

Residual plots showed that models using logFlux were consistently better than those using flux, regardless of whether MW or logP were used. The results will therefore focus on models utilising logFlux. Conversely, using K_p_ produced better predictive models for both physicochemical parameters than log K_p_, and so models presented use K_p_ not logK_p_.

When investigating the important predictors of logFlux, the effect of logP and MW were investigated individually, and top models for each selected (Table 1). Five MW-containing models and six logP-containing models were identified as top models. For MW models, all the top models allowed for heteroskedasticity of data by MW, whereas for logP models, allowing for heteroskedasticity in both logP and skin region produced the top models.

**Table 1:** 

**Models including logP**			
**Model**	**LogP**	**Weight**	**SubRegion**
F.logP-1	+	+	+
F.logP-4*	+		+
F.logP-9	X	X	X
F.logP-17	X	X	+
F.logP-25	+	X	X
F.logP-28	X		X
**Models including MW**			
**Model**	**MW**	**Weight**	**SubRegion**
F.MW-1	+	+	+
F.MW-6		+	+
F.MW-9	X	X	X
F.MW-17	X	X	+
F.MW-25	X	+	X

Top models for predicting logFlux in cane toads. +: additive effect, X: interaction.

*best model—used to predict values for experiments

Comparison of the logP- and MW-containing models showed that logP was consistently a better predictor of logFlux than MW. Indeed, even the worst-performing model containing logP as a predictor variable was significantly better than the best MW-containing model (L-ratio_6,8_ = 37.07598, p<0.0001). For the logP models, logP and skin region of application were found to be important predictors of logFlux, appearing in all of the top models. Animal weight was also important, but not essential, for better model fit, appearing in 5/6 of the top models. Molecular weight was not a good predictor of logFlux, as inclusion of MW in models decreased the model fit, however if MW was included in a model, animal weight became an important predictor variable for all models. Following comparison of goodness-of-fit and parsimony, F.logP-4 was selected as the top model and was used to predict flux values for the in vivo experiment. This model did not include weight as a predictive variable and allowed for heteroskedasticity in both logP and skin region of application.

When investigating the important predictors of K_p_, three top models for logP and four top models for MW were identified (Table 2). Interestingly, whereas allowing for heteroskedasticity in logP and skin region improved model fits for all logFlux models, the best K_p_ models including logP allowed for heteroskedasticity in logP only. Additionally, unlike the logFlux models where all logP-containing models consistently outperformed even the top MW-containing models, the top models were generally more similar, regardless of physicochemical parameter used. The exceptions were K.logP-9, which was significantly better than 50% of the MW models (models K.MW-17 and K.MW-25; L-ratio_16,10_ = 13.84304, p = 0.0314 and L-ratio_16,11_ = 12.08116, p = 0.0337 respectively), and K.logP-25, which also outperformed MW-17 (L-ratio_11,10_ = 5.986835, p = 0.0144).

**Table 2:** 

**Models including logP**			
**Model**	**LogP**	**Weight**	**SubRegion**
K.logP-9	X	X	X
K.logP-25	X	+	X
K.logP-28*	X		X
**Models including MW**			
**Model**	**MW**	**Weight**	**SubRegion**
K.MW-6		+	+
K.MW-9	X	X	X
K.MW-17	X	X	+
K.MW-25	X	+	X

Top models for predicting K_p_ in cane toads. +: additive effect, X: interaction.

*best model—used to predict values for experiments

For logP-containing models, both logP and skin region of application were important factors in predicting K_p_, appearing in all top models. Animal weight was also important, but not essential, for good model fit, appearing in 2/3 of the top logP models. Similar trends were seen in the MW models, with all three factors contributing to the top models. Following comparison of goodness-of-fit and parsimony, K.logP-28 was selected as the model to be used to predict K_p_ values for the in vivo experiment. This model did not include weight as a predictive variable and allowed for heteroskedasticity in logP only.

The final models of absorption, used for predictions of in vivo absorption parameters, are presented in Tables 3 and 4.

**Table 3:** 

Skin Region	Equation	Standard deviations	
		Intercept	Residual
Dorsal	*logFlux* = 1.869444−0.438706*LogP*	0.089362	0.059183
Thoracic	*logFlux* = 1.817442−0.438706*LogP*		
Pelvic	*logFlux* = 1.915197−0.438706*LogP*		

Equations to predict flux through different skin regions in *Rh*. *marina*. Model F.logP-4 was used to produce these equations.

**Table 4:** 

Skin Region	Equation	Standard deviations	
		Intercept	Residual
Dorsal	*K_p_* = 0.00318−0.000125*LogP*	0.000679	0.000552
Thoracic	*K_p_* = 0.003641−0.000302*LogP*		
Pelvic	*K_p_* = 0.004381−0.000373*LogP*		

Equations to predict permeability coefficient (K_p_) through different skin regions in *Rh*. *marina*. Model K.logP-28 was used to produce these equations.

### In vivo absorption of benzoic acid, caffeine, and ibuprofen

**Fig 1:**
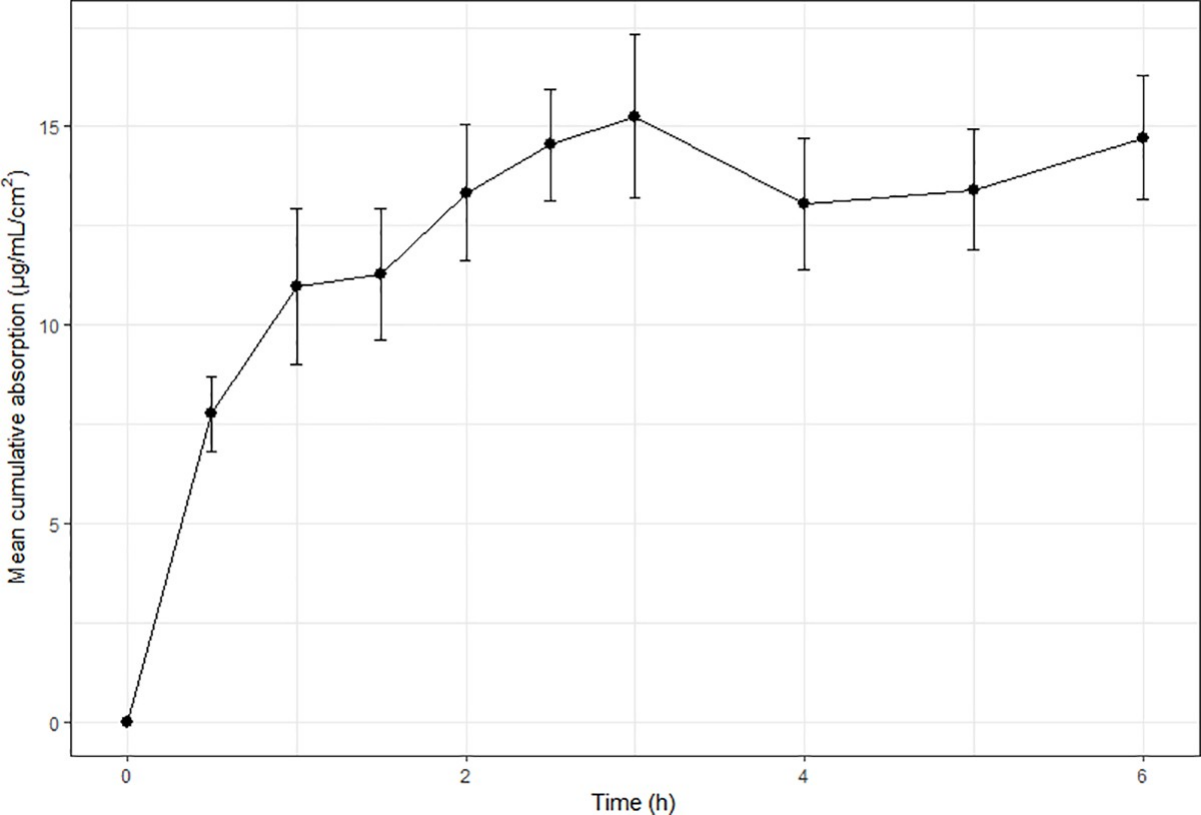
Mean cumulative absorption versus time curves for the in vivo absorption of benzoic acid in cane toads. Each in vivo data point represents the mean of four animals (N = 4) and error bars are SEM.

**Fig 2:**
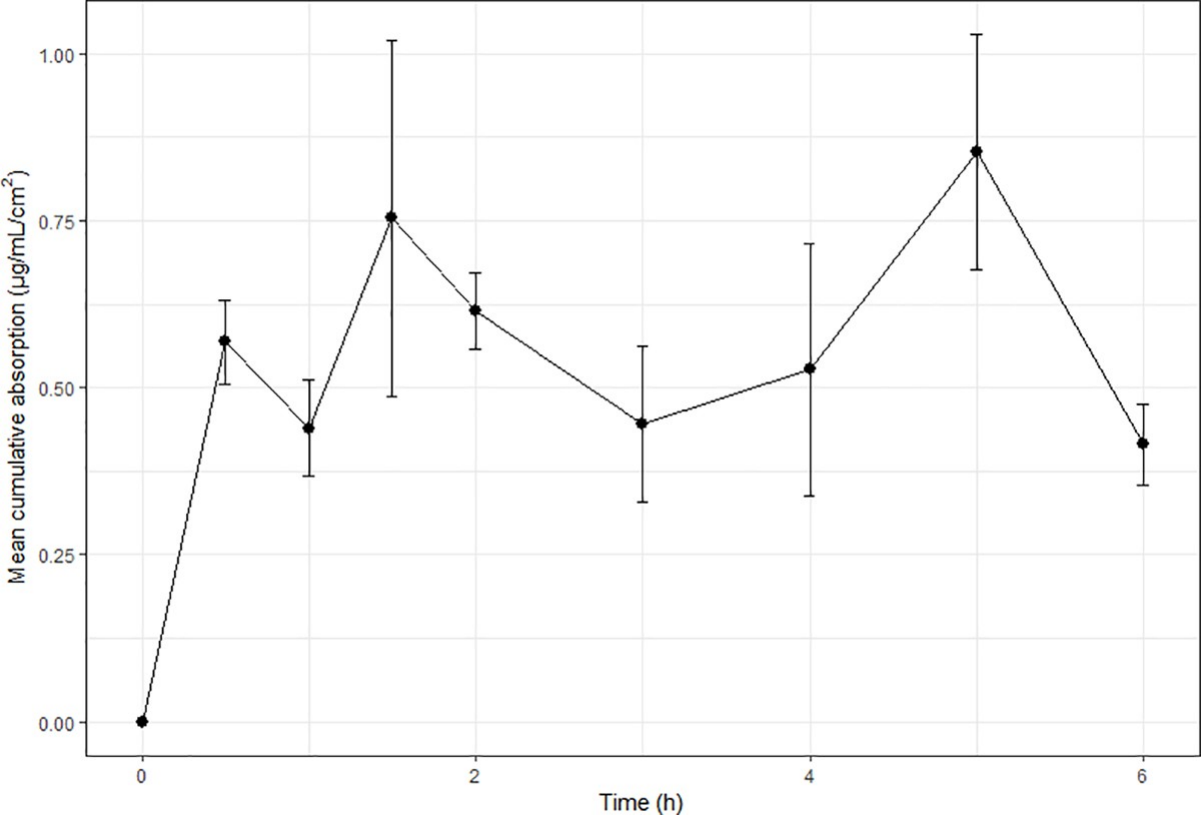
Mean cumulative absorption versus time curves for the in vivo absorption of ibuprofen in cane toads. Each in vivo data point represents the mean of four animals (N = 4) and error bars are SEM.

**Fig 3:**
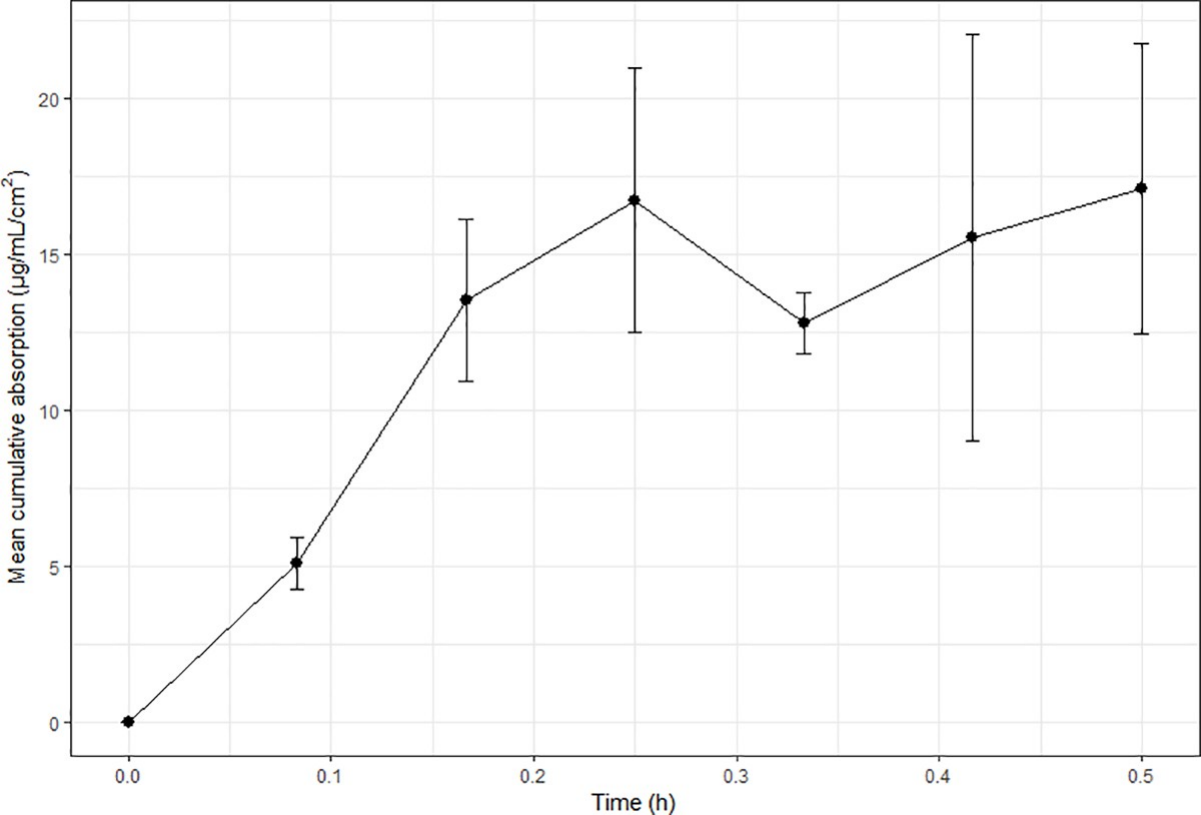
Mean cumulative absorption versus time curves for the in vivo absorption of caffeine in cane toads. Each in vivo data point represents the mean of four animals (N = 4) and error bars are SEM.

Benzoic acid absorption was the most consistent and least variable between frogs (Fig 1), with steady-state achieved at 90–120 minutes and maintained thereafter. In vivo ibuprofen levels increased over the first 30 minutes to 0.5687 μg/mL, and remained relatively constant for the remainder of the study (serum range over remaining sampling points: 0.415–0.852 μg/mL; Fig 2). Variability between individuals and sampling times changed in the ibuprofen study, being quite small at some sampling times, and large at others. No apparent trends in this variability were noted. Caffeine absorption was rapid up until t = 15 minutes (Fig 3), with a reduction in serum levels noted at the next sampling time (t = 20 minutes) indicative of elimination. Of note is that this reduction in serum levels at t = 20 minutes had the least inter-individual variability of all sampling times, suggesting relatively consistent absorption and elimination kinetics of caffeine in these animals.

### Predicted versus observed values of flux and K_p_ for benzoic acid, caffeine, and ibuprofen

Values of flux and K_p_, predicted using models F.logP-4 and K.logP-28, respectively, and as measured in the in vivo studies, are presented in Table 5. While the predicted values of flux and K_p_ were consistently higher than observed, the FOD between the predicted and observed values for K_p_ and flux, for each chemical, was very similar. The smallest FOD was seen for benzoic acid (4.7 for flux and 4.9 for K_p_), and the largest for ibuprofen (34.2 and 32.6 for flux and K_p_, respectively).

**Table 5:** 

Chemical	Predicted values		Observed values		Factor-of-difference	
	Flux^a^ (μg/cm^2^/h)	K_p_^b^ (x10^−3;^ cm/h)	Flux (μg/cm^2^/h)	K_p_ (x10^−3^; cm/h)	Flux	K_p_
Caffeine	88.289	4.408	7.181	0.359	12.295	12.279
Benzoic acid	12.440	3.684	2.653	0.744	4.689	4.952
Ibuprofen	1.491	2.901	0.044	0.089	33.886	32.596

Predicted (in vitro) and observed (in vivo) values, and factor-of-difference between them, for the flux and K_p_ three chemicals in pelvic ventral cane toad skin.

^a^: predicted using model F.logP-4

^b^: predicted using model K.logP-28

### Predicted and measured serum concentrations at conclusion of exposure time

Table 6 presents the predicted and observed serum concentrations for all three chemicals at their final sampling time. Predictions from both models were similar, with serum levels differing by no more than 4 μg/mL/cm^2^. All in vivo serum concentrations were lower than predicted, with the FOD ranging from ~2.58 (caffeine) to ~10.25 (ibuprofen). Interestingly, F.logP-4 more accurately predicted benzoic acid levels, whereas F.LogP-28 resulted in slightly lower FOD for caffeine and ibuprofen.

**Table 6:** 

Chemical	Serum concentration (μg/mL/cm^2^)			Factor-of-difference	
	Predicted F.logP-4	Predicted K.logP-28	Measured	F.logP-4	K.logP-28
Caffeine	44.14451	44.08441	17.106	2.580645	2.577131
Benzoic acid	74.6396	78.77866	14.724	5.069248	5.350357
Ibuprofen	8.947212	8.529969	0.852	10.50142	10.0117

Predicted serum concentrations from each of the top models, measured serum concentrations at the final sampling time, and factor-of-difference between predicted and measured values chemical

## Discussion

To determine the utility of extrapolating in vitro data to predict in vivo absorption following topical chemical exposure in frogs, the current study created models of absorption in the cane toad from in vitro absorption data, and then compared absorption kinetic values predicted by the model to in vivo absorption parameters measured in the same species.

In designing a model of percutaneous absorption in frogs, K_p_ and flux were both best-described by logP, not MW. Indeed, in the case of flux predictions, the top MW models performed worse than the poorest-fitting logP-based model. These results are perhaps unsurprising; the model drugs chosen in this study are recommended for use in percutaneous absorption studies because they represent a limited range of MW (122.12–206.29 Da), while providing a wide range in logP (–0.07 to 3.97). This is because in empirical models of percutaneous absorption in humans, logP has been found to be the primary determinant of absorption rate [25], with MW having a smaller role in regulating the absorption process. As frog skin is known to be permeable to large molecules including hormones [8], it is unsurprising that with the limited range of MW represented in the current model, MW did not significantly influence absorption. The impact of increasing MW on absorption rate in frog skin is a logical extension of the current study.

The models developed emphasise the importance of considering the site of chemical application in frogs, as all top models included both logP and skin region of application. In particular, ventral pelvic application increased absorption, especially for hydrophilic chemicals. Skin site is known to affect absorption in mammals, with regional variation in absorption being attributed to differences in skin thickness and follicle density. While frogs are hairless, there can be significant differences in skin thickness and vascularity between skin regions, in particular between the pelvic ventrum and other skin regions [26, 27]. The pelvic ventrum in terrestrial and arboreal frog species is a thin, highly vascularised region, allowing rapid uptake of water from underlying substrate. The ability of this region to permit rehydration differs between frog species, both from the same primary habitat and between habitats. Studies in cane toads have found heightened uptake of water through this region, particularly in dehydrated individuals [26, 28], despite reports of similar skin thickness between the dorsal and ventral surfaces. While absorption studies in frogs have traditionally investigated ventral versus dorsal surfaces, rarely is the ventral surface considered as two distinct regions for absorption. Visual inspection of the inner skin surface in toads shows a marked difference in the density and thickness of the capillaries on the ventral surface—being larger and more densely arranged in the pelvic region, and similar vascularity on the thoracic and dorsal skin surfaces (personal observation). Further, previous studies in cane toads by the authors [13] showed significantly higher absorption of a hydrophilic chemical through ventral pelvic skin compared to ventral thoracic (or dorsal) skin; it is therefore important to delineate between the ventral pelvic and ventral thoracic regions when applying chemicals to the skin of frogs, to minimise inadvertent over- or under-dosing.

The current study showed consistently lower in vivo values than predicted from in vitro observations. This is consistent with others studies using diffusion cells to predict in vivo absorption in frogs [11, 17], and has also been reported in mammalian studies [5, 6, 29]. Additionally, despite this finding of lower overall absorption in vivo, all chemicals demonstrated similar or more rapid initial in vivo absorption. The most extreme was benzoic acid, which exhibited a 50% higher concentration in vivo at the initial sampling time compared to previously-reported in vitro levels [13], whereas in vivo ibuprofen levels were marginally lower at t = 30 minutes than the reported in vitro levels. Such a reduction in the overall in vivo levels compared to predicted or observed in vitro values is not unexpected, as in vitro experiments represent an isolated process (absorption), whereas in vivo there are competing processes of elimination and distribution occurring concurrently with absorption. This was obviously a contributing factor in the current study, as all three chemical absorption profiles appear to have reached steady-state (Figs 1–3), and most animals urinated during the study. In order to deconvolute the in vivo data, IV elimination pharmacokinetic data would be required. However, such data are rare in frogs, and does not exist for the chemicals studied. There have been attempts to convert pharmacokinetic elimination data from other species (via allometric and/or relative metabolic capacity) to apply to frogs [30], however results were variable between chemicals studied, and for different frog species. Additionally, elimination pathways are likely to be different between mammals and frogs, owing to known differences in microsomal enzyme activities and renal function [31, 32]. Thus, it is inappropriate to use data from mammalian species in the current study to estimate elimination kinetics.

Despite the lower in vivo absorption values reported in the current study, reasonable correlation was found between predicted absorption rates and observed in vivo absorption parameters for benzoic acid and caffeine. Indeed, the FOD reported for benzoic acid and caffeine in the current study are lower and similar to (respectively) those reported by Riviere, Shapiro [11], who suggested that these levels provided a reasonable correlation, given the inherent difficulties with accurately extrapolating in vitro data to the in vivo situation. In addition to the masking effects of concurrent elimination processes occurring in vivo, the assumption that flux is constant from the start of dosing when predicting serum concentrations is another potential source of error. A lag-phase has only been reported in frog skin in vitro when the donor solution contains penetration enhancers [33]; as the first sample in the current study was taken at 30 minutes, it is possible that the lag phase occurred prior to this sample being taken. Disregarding even a short lag-time when predicting serum concentration would contribute to the disparity between in vitro predictions and in vivo concentrations. Finally, while an attempt has been made to estimate the surface area of skin exposed to the bathing solution, extrapolation of dosing area from each animal’s weight is likely to have contributed to the differences between in vitro predictions and in vivo observations in the current study.

Studies examining in vitro and in vivo absorption in mammals commonly report poorer agreement between in vitro and in vivo values for hydrophilic chemical absorption [5, 29], whereas the current study found the worst predictive power for ibuprofen, the most lipophilic chemical. Despite these seemingly contradictory results, this difference can be explained by the differences in skin composition and physiology between these animals. The stratum corneum in mammals is a thick, largely lipophilic structure, and as such, retards absorption of hydrophilic substances. However, the reverse is true in frogs when applying substances to the ventral pelvic skin—an area honed for uptake of water—and so lipophilic substances have reduced absorption. Additionally, substances absorbed to a lesser degree will result in an amplification in any interindividual variability in pharmacokinetic processes [34], and so it is unsurprising that lipophilic ibuprofen exhibited the poorest agreement between in vitro and in vivo absorption. Although within dermal absorption testing guidelines for sample sizes [20], the use of only four animals per sampling time may have also contributed to these findings–as the interindividual variability would be more pronounced with fewer individuals contributing to the data point.

Finally, it should also be noted that any urine excreted would have been added to the bathing solution, which would make the chemical available for reabsorption, while also slightly diluting the bathing solution. This dilution of (and the addition of excreted chemical into) the bathing solution is unlikely to impact absorption rate significantly as the bathing solution was saturated with chemical, and so thermodynamics of the solution are unlikely to be significantly affected by these small changes in chemical and bathing fluid levels. However, accumulation of drug in the urine would reduce serum concentrations. Studies to ascertain baseline pharmacokinetic elimination parameters for each model chemical will permit more detailed analysis of the data presented herein.

## Conclusions

Both models presented provide reasonable predictions of serum concentrations in vivo, particularly for moderately lipophilic and hydrophilic chemicals, and emphasise the importance of considering both application site and physicochemical properties when considering percutaneous absorption in frogs. However, similar to the seminal work off Franz [6] on the relevance of in vitro studies to in vivo human percutaneous absorption, although the findings of the current study suggest a moderate agreement between in vitro and in vivo absorption in frogs, further work is needed to produce more harmonious absorption data to refine the model.

In particular, investigations into the in vivo pharmacokinetics, including IV administration, of these model chemicals will permit deconvolution of the data and offer the ability to separate the absorption phase from elimination. Another logical step would be to further investigate the utility of this model by using it to predict the serum concentration following topical administration of a chemical for use in the treatment of disease in frogs, and to then investigate and compare the in vivo absorption of this chemical with the predictions. It is likely that the findings herein will provide a useful method to estimate the in vivo absorption of hydrophilic and moderately-lipophilic chemicals through frog skin, and could therefore be used in predicting absorption when formulating chemicals for treatment of disease in frogs.
